# Humans as blood-feeding sources in sylvatic triatomines of Chile unveiled by next-generation sequencing

**DOI:** 10.1186/s13071-023-05841-x

**Published:** 2023-07-06

**Authors:** Esteban San Juan, Raúl Araya-Donoso, Catalina Sierra-Rosales, Juana P. Correa, Nicol Quiroga, Ricardo Campos-Soto, Aldo Solari, Martin Llewellyn, Antonella Bacigalupo, Carezza Botto-Mahan

**Affiliations:** 1 Departamento de Ciencias Ecológicas, Facultad de Ciencias, Universidad de Chile, Santiago, Chile; 2 School of Life Sciences, Arizona State University, Tempe, AZ USA; 3 Facultad de Ciencias de la Naturaleza, Universidad San Sebastián, Concepción, Chile; 4 Escuela de Ciencias Agrícolas y Veterinarias, Universidad Viña del Mar, Viña del Mar, Chile; 5 Programa de Biología Celular y Molecular, Facultad de Medicina, Instituto de Ciencias Biomédicas, Universidad de Chile, Santiago, Chile; 6 School of Biodiversity, One Health and Veterinary Medicine, University of Glasgow, Glasgow, UK

**Keywords:** *Mepraia spinolai*, *Mepraia parapatrica*, *Triatoma infestans*, Chagas disease, NGS, Chile

## Abstract

**Background:**

Triatomines are blood-sucking insects capable of transmitting *Trypanosoma cruzi*, the parasite that causes Chagas disease in humans. Vectorial transmission entails an infected triatomine feeding on a vertebrate host, release of triatomine infective dejections, and host infection by the entry of parasites through mucous membranes, skin abrasions, or the biting site; therefore, transmission to humans is related to the triatomine–human contact. In this cross-sectional study, we evaluated whether humans were detected in the diet of three sylvatic triatomine species (*Mepraia parapatrica*, *Mepraia spinolai*, and *Triatoma infestans*) present in the semiarid–Mediterranean ecosystem of Chile.

**Methods:**

We used triatomines collected from 32 sites across 1100 km, with an overall *T. cruzi* infection frequency of 47.1% (*N* = 4287 total specimens) by conventional PCR or qPCR. First, we amplified the vertebrate cytochrome b gene (*cytb*) from all DNA samples obtained from triatomine intestinal contents. Then, we sequenced *cytb*-positive PCR products in pools of 10–20 triatomines each, grouped by site. The filtered sequences were grouped into amplicon sequence variants (ASVs) with a minimum abundance of 100 reads. ASVs were identified by selecting the best BLASTn match against the NCBI nucleotide database.

**Results:**

Overall, 16 mammal (including human), 14 bird, and seven reptile species were identified in the diet of sylvatic triatomines. Humans were part of the diet of all analyzed triatomine species, and it was detected in 19 sites representing 12.19% of the sequences.

**Conclusions:**

Sylvatic triatomine species from Chile feed on a variety of vertebrate species; many of them are detected here for the first time in their diet. Our results highlight that the sylvatic triatomine–human contact is noteworthy. Education must be enforced for local inhabitants, workers, and tourists arriving in endemic areas to avoid or minimize the risk of exposure to Chagas disease vectors.

**Graphical Abstract:**

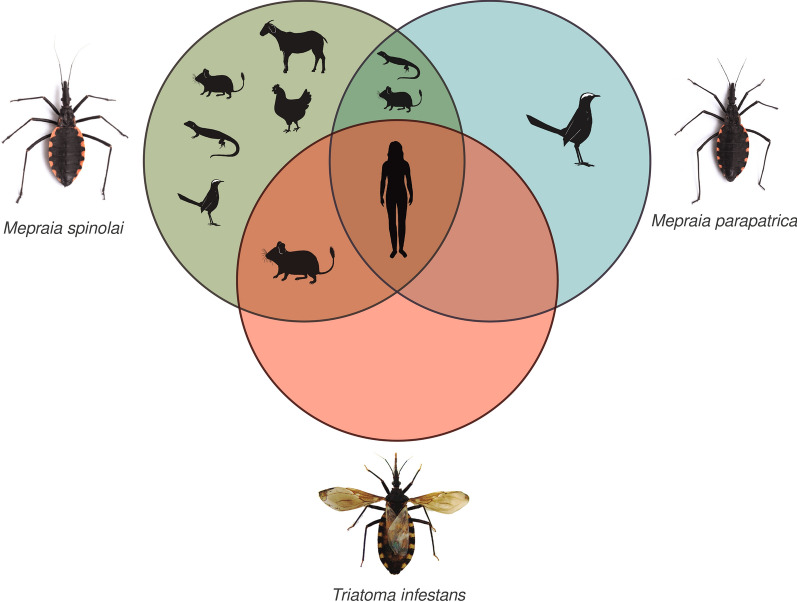

**Supplementary Information:**

The online version contains supplementary material available at 10.1186/s13071-023-05841-x.

## Background

The study of the dietary composition in sylvatic vectors of zoonotic pathogens is particularly relevant for public health because it provides indirect evidence about the host species involved in the maintenance of endemic vector-borne infections [1–3]. Triatomines (Hemiptera: Reduviidae: Triatominae) are hematophagous vector species, commonly known as kissing bugs, capable of transmitting *Trypanosoma cruzi* (Kinetoplastea: Trypanosomatidae), the parasite that causes Chagas disease [4]. The transmission cycle involves feeding of an infected triatomine on a vertebrate host species, the release of its infective dejections, and the posterior parasitic infection of the host’s mucous membranes, skin abrasions, or the biting site [5]. In addition, oral transmission has been described as an important route of infection, by accidental consumption of insects or their feces in humans, by entomophagy in insectivorous mammals, or as a defensive measure against the triatomine bite in other mammal species [6]. Therefore, transmission to humans is related to the triatomine–human contact frequency [7, 8].

In north-central Chile, there are four described triatomine species, all reported as infected with *T. cruzi*: the sylvatic diurnal species *Mepraia spinolai*, *Mepraia gajardoi*, and *Mepraia parapatrica*, and the nocturnal species *Triatoma infestans* [9–12]. Additionally, 25 mammal species (14 native and 11 exotic) and four lizard species have also been described infected with *T. cruzi* [12–15]. The main sylvatic vector of *T. cruzi* (between ~ 26.5° and 34° S) is *M. spinolai* [16, 17], an abundant species whose preferred microhabitats include rocky outcrops, rock piles, burrows, and bromeliads, but it has also been found near human settlements and inside rural houses [18–22]. Along its geographical distribution, *M. spinolai* populations show *T. cruzi* infection prevalence ranging from 1.3% to 99.0% [22]. However, prevalence and population abundance vary temporally and spatially depending on the local ecological characteristics of the prospected sites (e.g., vertebrate composition) and climatic features [17, 22–24]. *Mepraia spinolai* feeds mostly on native mammals and birds [25–29]. The native rodents *Octodon degus* and *Phyllotis darwini* have been found infected by *T. cruzi* and they are described as the most important feeding sources of *M. spinolai* [26–30, 17]. However, inside houses, humans are the most frequently detected feeding source, followed by domestic animals [31]. Most previous studies have assessed the feeding profile of *M. spinolai* in a few specific localities, targeting specific host species in the search process and, therefore, describing only a part of the whole spectrum of vertebrate species included as prey throughout its distribution.

*Mepraia gajardoi* and *M. parapatrica* inhabit coastal and insular areas between 18° and 26° S [16, 32, 33]. Both species present low population abundance and can be found under rocks and associated with fishermen’s dwellings. Their *T. cruzi* infection prevalence ranges from 5.8 to 71.4%, depending on the prospected location [10, 11, 32, 34, 35]. A serological study showed that on the Pan de Azúcar island (26°09′ S, 70°39′ W), specimens of *M. parapatrica* had fed on marine birds (78%), sea mammals (15%), and reptiles (7%) [36], but not on humans. Moreover, a recent DNA-based detection study carried out on the same island and nearby coastal locations, showed that 61.3% of the blood meal sources corresponded to two lizard species (*Microlophus atacamensis* and *Garthia gaudichaudii*), 35.5% to three mammal species (the rodents *Mus musculus* and *Abrothrix olivaceus*, and human), and 3.2% to one bird species (the vulture *Cathartes aura*) [35]. These results highlight the importance of testing for the participation of humans in the kissing bugs’ diet in other localities where these species are occurring, especially near human settlements.

*Triatoma infestans* used to be associated with the domestic cycle of transmission of *T. cruzi*, due to the widespread colonies inside houses across the country, where it fed on humans and domestic animals. Currently, this species has been controlled by a sustained campaign of residual insecticide spraying in human dwellings, and mainly flying adults are reported invading houses within the endemic area of Chagas disease [37]. Domiciliary findings of this species show between 20 and 70% of *T. cruzi* infection [38]. The origin of these individuals is supposed to be sylvatic foci, and they have been reported in bromeliads and rock piles [19, 20, 39]. Infection by *T. cruzi* in these sylvatic specimens varies, with reports ranging from 25 to 41% [12, 19, 20]. Despite the relevance of studying blood-feeding sources of sylvatic *T. infestans*, there is no published information describing their diet in Chilean foci.

*Trypanosoma cruzi* infection has been detected mainly in people from rural and suburban areas of Chile, between 18°30′ and 34°16′ S [40]. Chagas disease prevalence in children and house infestation rates have dropped substantially in the past two decades [41]. Even though Chilean intradomiciliary vector-borne transmission of *T. cruzi* by *T. infestans* was declared interrupted in 1999, sylvatic triatomine vectors are still an important problem in rural areas [38, 41, 42], which has maintained the endemicity of the disease in the country. Thus, the description of the whole spectrum of natural hosts should be a priority in public health programs, especially considering the current human infection prevalence of 1.2% by immunoglobulin G (IgG) seropositivity and that ~ 800,000 people are at risk of infection in Chile [43].

Most studies reporting the feeding profile of triatomines have used immunological techniques (e.g., enzyme-linked immunosorbent assay [ELISA]), and only in the last decade DNA-based identification of blood meal sources (e.g., high-resolution melting, Sanger sequencing, next-generation sequencing [NGS]) has been implemented to describe the diet of these vectors [28, 29, 35, 44–49]. In the present study, we use DNA-based blood meal source detection, aiming to (i) identify the vertebrate species included in the diet of sylvatic triatomines from most endemic areas of Chile, and (ii) evaluate the frequency of humans in the diet. This information will characterize for the first time the whole spectrum of vertebrate species acting as prey of sylvatic triatomines, provide an indirect triatomine–human contact frequency, and report the potential vertebrate species involved in the transmission cycle and maintenance of *T. cruzi* in Chile.

## Methods

### Study sites and triatomine samples

**Fig. 1:**
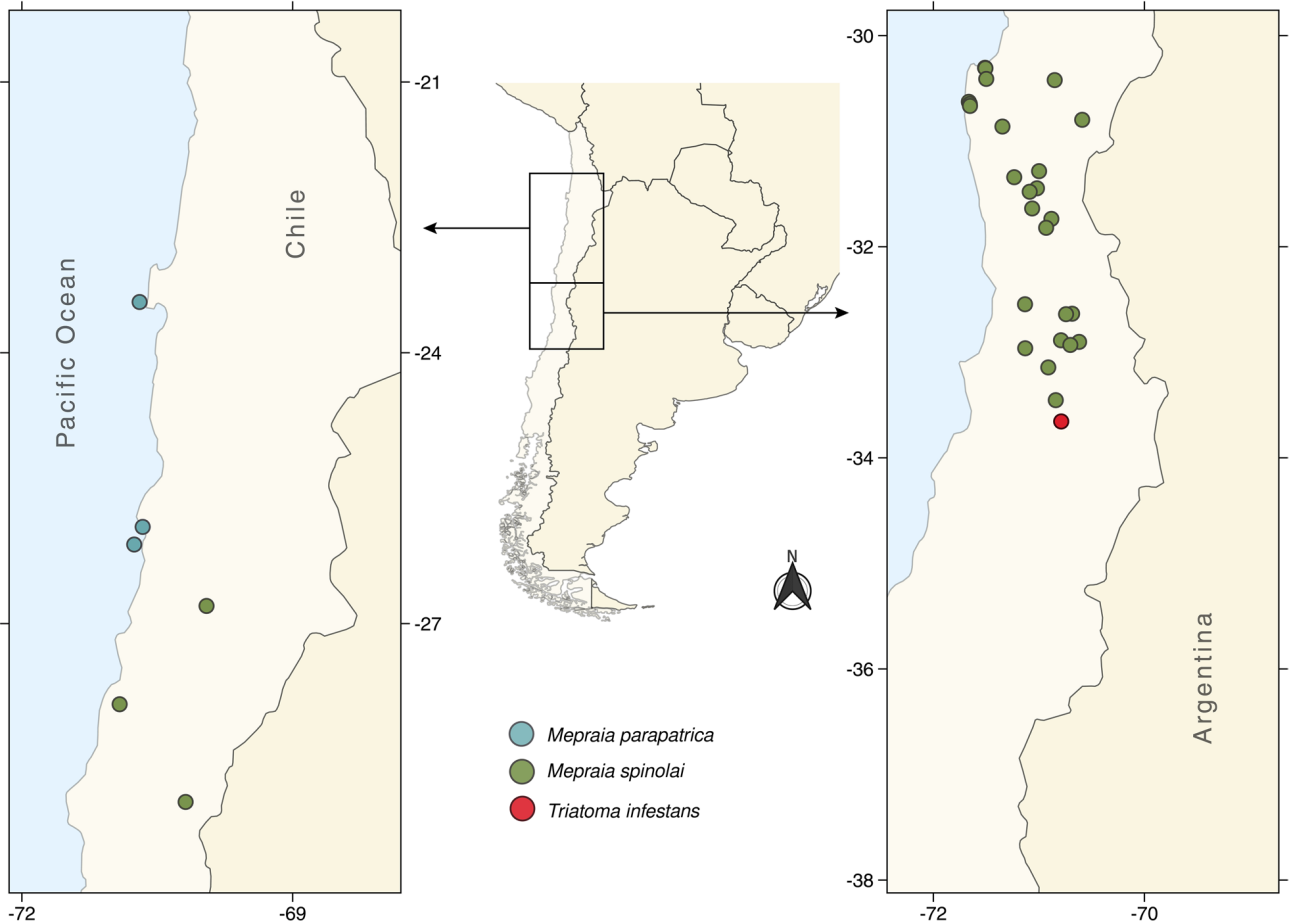
Location of the study sites with presence of sylvatic triatomines in north-central Chile, South America. Blue circles of *Mepraia parapatrica* include one *Mepraia* sp. population. Authors’ own map produced using QGIS

In this cross-sectional study, we assessed the feeding profile of 32 sylvatic triatomine populations, across 1100 km in the arid-semiarid-Mediterranean ecosystems of the Pacific side of southern South America, Chile (23°25′ to 33°26′ S; Fig. 1). Triatomines were collected during summer, from 2014 to 2020. Twenty-one populations were analyzed as part of previous research studies (18 from *M. spinolai* [*N* = 2992], two from *M. parapatrica* [*N* = 87] and one *Mepraia* sp. population [*N* = 38]; sample size per population is shown in Additional file 1: Table S1 [22, 32, 35]), and 11 populations were sampled/analyzed for the present study (10 from *M. spinolai* [*N* = 1116] and one from *T. infestans* [*N* = 54], sample size per population is shown in Additional file 1: Table S1). In each study site, *Mepraia* specimens were manually collected for 3–5 days during the daytime (10:00–18:00) by trained researchers using appropriate clothing to avoid triatomine–human contact. *Triatoma infestans* were collected in a single site, during one night (19:00–09:00), using 85 yeast-baited traps placed underneath bromeliads [12]. Collected specimens were individually stored and frozen at −20 °C until processing.

Sampling procedures were authorized by the Corporación Nacional Forestal (CONAF) from the Atacama and Coquimbo regions (permit numbers: 049/2017, 150/2017, 01/2018, 095/2018). This study was conducted in accordance with the guidelines established by the Institutional Committee for the Care and Use of Animals, University of Chile, Chile (permit number: 17074-FCS-UCH).

### DNA extraction of triatomine intestinal content

Triatomine samples collected for the present study were subjected to abdominal extrusion on a clean working area to obtain both intestinal content and intestine samples. An aliquot of a maximum of 25 mg of each sample was mixed with 20 μl of commercial nuclease-free water (Invitrogen™ UltraPure™ Dnase/Rnase-Free Distilled Water).

Whole DNA was isolated from the samples using the DNeasy^®^ Blood & Tissue Kit (QIAGEN, CA, USA). An internal amplification control (IAC) was added to each sample to assess the presence of inhibitors, consisting of 100 pg of *Arabidopsis thaliana* DNA [50]. The manufacturer's recommendations were followed with a few modifications: the samples were centrifuged for 4 min at 17,000 × *g* to dry the DNeasy Blood & Tissue Mini spin column, and the final elution volume was 100 μl. Samples were stored at −20 °C until molecular analysis. Triatomine samples from previous studies were processed, as previously described in [22, 32, 35] (Additional file 1: Table S1).

### *Trypanosoma cruzi* infection in sylvatic triatomine populations

*Trypanosoma cruzi* infection in triatomine samples obtained for this study was tested in a QuantStudio^®^ 3 real-time polymerase chain reaction (PCR) system (Thermo Fisher, USA). All samples were analyzed in duplicate using 0.4 µM of *T. cruzi* nuclear satellite DNA primers Cruzi 1 and Cruzi 2 [51], 1× HOT FIREPol^®^ EvaGreen^®^ qPCR [quantitative PCR] Mix Plus (Solis BioDyne, Tartu, Estonia), and 5 µl of DNA template in a final volume of 20 µl. DNA from two different *T. cruzi* strains (TCI-CYC and TCII-CDMC, kindly provided by Dr. Gonzalo Cabrera, Institute of Biomedical Sciences, Faculty of Medicine, University of Chile, Chile) were used as positive control and water instead of DNA as a no-template control. Cycling conditions were 15 min at 95 °C followed by 50 cycles at 95 °C for 15 s, 65 °C for 20 s, and 72 °C for 20 s, finishing with a default melting curve. To detect false negatives, a real-time PCR assay was conducted amplifying IAC DNA using IAC primers with a concentration of 0.4 µM [52] and the same qPCR mix previously described. The PCR conditions were 12 min at 95 °C followed by 40 cycles at 95 °C for 15 s, 64 °C for 15 s, and 72 °C for 15 s, finishing with a default melting curve. Individuals were considered infected when *T. cruzi* and IAC amplified, and the cycle threshold value (C_t_) was lower than 42. In addition, positive samples with a C_t_ higher than 42 were corroborated by electrophoresis, searching for the expected 166-base-pair (bp) amplicon [51]. For samples from previous studies, *T. cruzi* infection was detected as previously described in [22, 32, 35], where 121/122 or Cruzi 1/Cruzi 2 primers were used depending on the sampled population (see details in Additional file 1: Table S1).

### Vertebrate cytochrome b (*cytb*) DNA detection in triatomine samples

To assess the feasibility of blood meal detection, we performed a real-time PCR to detect the presence of *cytb* DNA in the triatomine samples from 30 populations (N = 4200 specimens). For the other two populations (N = 87 specimens), this assay was performed as described in [35]. In Additional file 1, Table S1, the sample size analyzed per population is indicated (see total number of captures). Vertebrate *cytb* DNA detection was carried out in a QuantStudio^®^ 3 Real-Time PCR System (Thermo Fisher, USA). First, samples were analyzed in duplicate using 0.5 µM of vertebrate *cytb* gene primers that amplify a 383-bp fragment [53], 1× HOT FIREPol^®^ EvaGreen^®^ qPCR Mix Plus (Solis BioDyne, Tartu, Estonia), and 2 µl of template in a final volume of 20 µl. Each assay included European rabbit (*Oryctolagus cuniculus*) DNA as positive control, and water instead of DNA as no-template control. Cycling conditions were 15 min at 95 °C followed by 40 cycles at 95 °C for 15 s, 60 °C for 20 s, and 72 °C for 20 s, finishing with a default melting curve. The samples with a C_t_ value below 34 were considered feasible for blood meal identification by NGS.

### Diet detection by NGS and bioinformatics

For 30 populations, we performed NGS using a pool of 10 to 20 blood meal samples of randomly chosen triatomine specimens for each population, in which vertebrate *cytb* DNA had been previously detected. We included *T. cruzi* infected and uninfected triatomines in equal proportions, when possible. In the other two populations, NGS was performed as described in [35] (Additional file 1: Table S1). We performed a diversity assay using bTEFAP^®^ Illumina 20 k on *cytb* of vertebrates, using the same primers described before [53]. The demultiplexed reads were filtered using Sickle 1.33 (https://github.com/ucdavis-bioinformatics/sickle; accessed on 28 October 2021), removing reads shorter than 200 bp or with a quality score lower than Q30. We removed adapter and primer sequences with CutAdapt 1.18 [54]. We then used DADA2 [55] to generate amplicon sequence variants (ASVs). ASVs with less than 100 reads of abundance were removed [56]. Then, each ASV was compared against *cytb* sequences from the National Center for Biotechnology Information (NCBI) database, with the BLASTn tool, available at https://blast.ncbi.nlm.nih.gov/Blast.cgi. Each ASV was assigned to the species corresponding to the highest blast score.

### Data processing and analysis

Blood-feeding sources for each triatomine species were obtained by adding the NGS results from all populations corresponding to that species (i.e., populations from previous studies and those from the present study). Additionally, we added the previously reported NGS diet detection data from two populations of *M. parapatrica* [35]. The feeding sources were classified into six groups: (i) human, considered independently because of its public health relevance; (ii) human-associated mammals, including livestock (e.g., goats, pigs), pets (e.g., dogs), and synanthropic rodents (e.g., rats, mice); (iii) mammals not associated with humans, which included native mammals, as well as introduced free-ranging mammals (e.g., the European rabbit, European hare); (iv) human-associated bird species; (v) birds not associated with humans; and (vi) reptiles.

## Results

### Sylvatic triatomine populations and *T. cruzi* infection

We studied 28 *M. spinolai* populations (*N* = 4108 specimens; distance to human settlements: range 59–4574 m and mean ± SD = 746 ± 933 m), two *M. parapatrica* populations (*N* = 87 specimens; distance to human settlements: range 45–2143 m and mean ± SD = 1094 ± 1484 m), one *Mepraia* sp. population (*N* = 38 specimens; distance to human settlement: 1285 m), and one sylvatic *T. infestans* population (*N* = 54 specimens; distance to human settlement: 5 m) (Fig. 1). Given that the *Mepraia* sp. population was geographically near to the two *M. parapatrica* populations [33], and all of them were coastal populations from islands or the continent, they were combined for the diet analysis. *Trypanosoma cruzi* infection frequency ranged from 0.0 to 99.0% for *M. spinolai*, 20.7 to 36.8% for *M. parapatrica*, and 55.6% for *T. infestans*. Details for each population are compiled in Additional file 1: Table S1.

### Diet by triatomine species

**Fig. 2:**
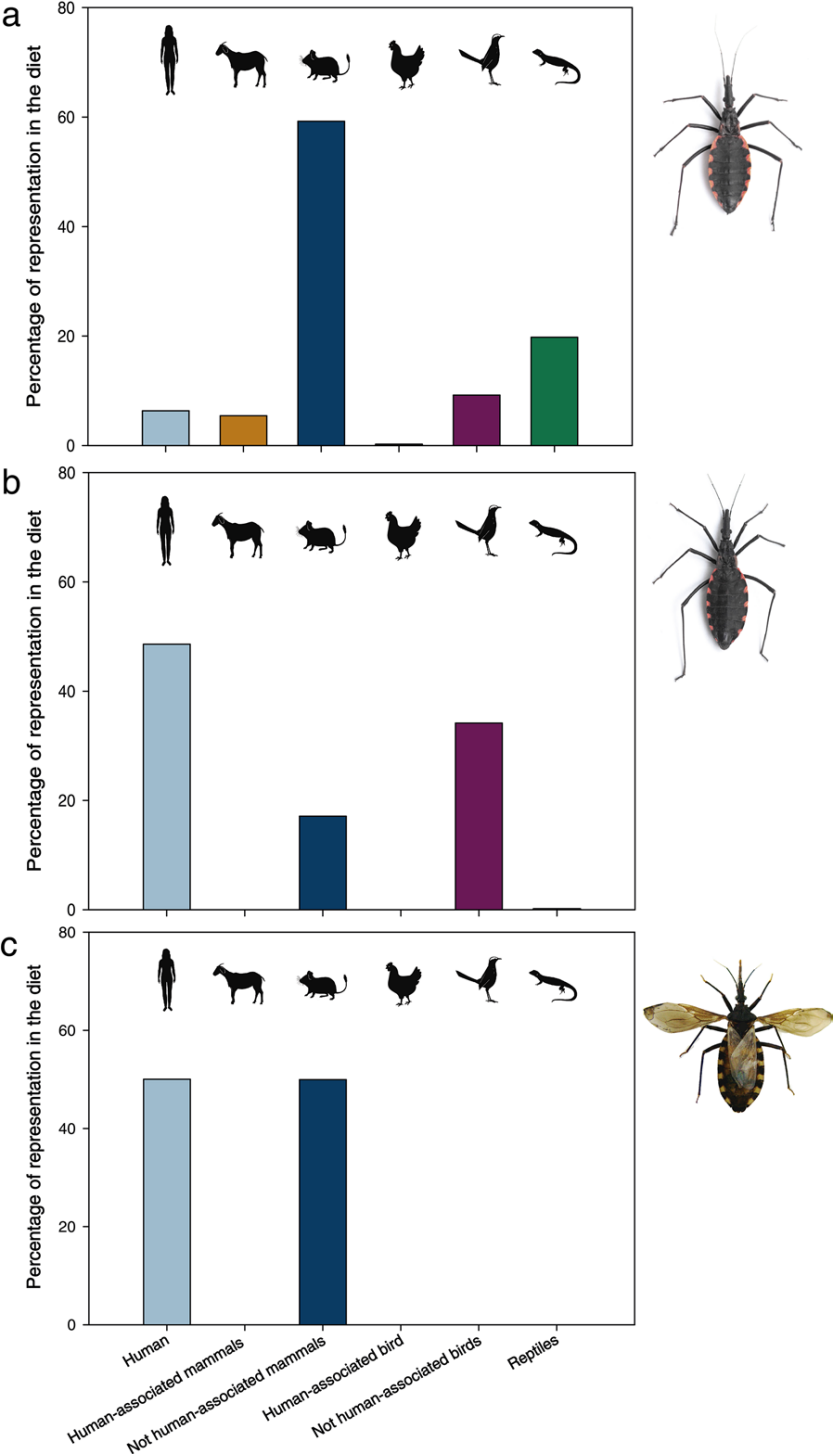
Percentage of vertebrate groups in the diet of **a** *Mepraia spinolai*, **b** *Mepraia parapatrica*, and **c** *Triatoma infestans* calculated as the number of reads of each vertebrate group over the total number of reads for each specific triatomine species determined by NGS

Overall, we obtained between 30,654 and 78,238 reads in the 32 sylvatic triatomine populations. Thirty-seven vertebrate species were identified as feeding sources for all triatomine species combined, including human, human-associated mammals (5), not human-associated mammals (10), human-associated bird (1), not human-associated birds (13), and reptiles (7) (see complete list of ASV assignations in Additional file 2: Table S2). For all populations of *M. spinolai*, the blood-feeding sources included 36 vertebrate species: human, human-associated mammals (5), not human-associated mammals (10), human-associated bird (1), not human-associated birds (12), and reptiles (7) (Fig. 2a). For *M. parapatrica* populations, a total of four vertebrate species were detected (human, one mammal, one bird, and one reptile), and no human-associated species was detected (Fig. 2b). Finally, the blood-feeding sources of *T. infestans* included only three vertebrate species (human and two not human-associated mammals) (Fig. 2c). These three wild triatomine species shared only one blood-feeding source in their diet: the human (*Homo sapiens*). A complete list of the vertebrate species detected as blood-feeding sources per triatomine species is included in Table 1 and per triatomine population is shown in Additional file 3: Table S3.

**Table 1:** Vertebrate species detected by NGS as a blood-feeding source of each sylvatic triatomine species shown by group classification

Triatomine species	Group	Order	Scientific name	% diet
*M. parapatrica*	H	Primates	*Homo sapiens*	48.46
	B	Accipitriformes	*Cathartes aura*	34.31
	M	Didelphimorphia	*Thylamys elegans*^a^	17.09
	R	Squamata	Gekkota sp.	0.14
*M. spinolai*	M	Rodentia	*Abrocoma bennettii*	18.04
	M	Rodentia	*Phyllotis darwini*	13.21
	M	Rodentia	*Octodon degus*	12.82
	M	Rodentia	*Octodon lunatus*^a^	7.39
	R	Squamata	*Liolaemus fuscus*^a^	7.06
	H	Primates	*Homo sapiens*	6.47
	R	Squamata	Gekkota sp.	5.20
	M	Lagomorpha	*Oryctolagus cuniculus*	4.77
	R	Squamata	*Liolaemus monticola*^a^	4.06
	M	Rodentia	*Phyllotis limatus*^a^	2.94
	B	Passeriformes	Tyrannides sp.	2.42
	R	Squamata	Iguanidae sp.	2.14
	MAH	Carnivora	*Canis lupus familiaris*	2.02
	MAH	Rodentia	*Mus musculus*	1.87
	B	Passeriformes	*Sicalis olivascens*^a^	1.69
	MAH	Artiodactyla	*Capra hircus*	1.61
	B	Passeriformes	*Phrygilus alaudinus*^a^	1.47
	B	Passeriformes	Icteridae sp.	1.38
	R	Squamata	*Liolaemus nitidus*	0.82
	B	Passeriformes	Turdidae sp.	0.78
	B	Passeriformes	*Diuca diuca*^a^	0.77
	B	Passeriformes	*Agriornis lividus*^a^	0.31
	B	Passeriformes	*Pteroptochos* sp.^a^	0.27
	B	Accipitriformes	*Vultur gryphus*^a^	0.12
	R	Squamata	*Liolaemus platei*	0.08
	R	Squamata	Pleurodonta sp.	0.06
	M	Rodentia	Sigmodontidae sp.	0.05
	B	Galliformes	*Callipepla californica*^a^	0.05
	MAH	Artiodactyla	*Sus scrofa*^a^	0.03
	M	Lagomorpha	*Lepus europaeus*^a^	0.02
	MAH	Rodentia	*Rattus rattus*	0.02
	B	Passeriformes	*Muscisaxicola rufivertex*^a^	0.01
	B	Passeriformes	*Pseudasthenes humicola*^a^	0.01
	M	Didelphimorphia	*Thylamys elegans*	0.01
	M	Rodentia	*Abrothrix olivaceus*	0.01
	BAH	Galliformes	*Gallus gallus*	0.01
*T. infestans*	H	Primates	*Homo sapiens*	50.69
	M	Rodentia	*Octodon degus*^a^	38.01
	M	Lagomorpha	*Oryctolagus cuniculus*	11.30

*H* humans, *MAH/M* mammals associated/not associated to humans, *BAH/B* birds associated and not associated to humans, *R* reptiles. The percentage of each vertebrate species in the diet was obtained from the number of reads for that specific vertebrate over the total reads of vertebrates within each triatomine species

^a^First detection in the diet of that sylvatic triatomine species in Chile

When considering the read abundance for each vertebrate group detected as blood-feeding sources for the three species combined, humans represented 12.19%; human-associated mammals, 4.81%; not human-associated mammals, 54.67%; human-associated birds 0.01%, not human-associated birds, 11.52%; and reptiles, 16.81%. When analyzing the feeding sources of each triatomine species separately, the most represented vertebrate group was not human-associated mammals for *M. spinolai* (59.28%), and human for *M. parapatrica* and *T. infestans* (48.46% and 50.69%, respectively) (Fig. 2). For *M. spinolai*, human DNA represented 6.47%.

The three most represented species in the diet of *M. spinolai* were the mammals *Abrocoma bennettii* (18.04%), *P. darwini* (13.21%), and *O. degus* (12.82%). Interestingly, species such as moon-toothed degu (*O. lunatus*), human (*H. sapiens*), rabbit (*O. cuniculus*), and three reptiles (*Liolaemus fuscus,* Gekkota sp., and *L. monticola*) were also represented in a high proportion (see details in Table 1). For *M. parapatrica*, the three most important species in the diet were *H. sapiens* (48.46%), *C. aura* (34.31%), and *Thylamys elegans* (17.09%). Finally, in *T. infestans*, the only three species present in the diet were *H. sapiens* (50.69%), *O. degus* (38.01%), and *O. cuniculus* (11.30%).

## Discussion

In the last decade, DNA-based identification of blood meal sources has been implemented to describe the diet of triatomine species. In this study, we used NGS to identify the vertebrate species included in the diet of three sylvatic triatomine species present in the semiarid-Mediterranean ecosystem of Chile and evaluated the frequency of humans as their blood-feeding source.

The blood-feeding sources of *M. spinolai* included the six established groups, with mammals not associated with humans being the most represented group. Several species of wild mammals are known to play crucial roles in the maintenance of *T. cruzi* in the Chilean semiarid-Mediterranean ecosystem due to their infection frequencies [12, 17], and this group of vertebrates has been reported as the most frequent and abundant blood meal source in *M. spinolai* populations, with *O. degus* and *P. darwini* as the most common [26–28]. In our study, the most represented species was *A. bennettii*, which had not been previously described as an important blood source; however, this large-sized rodent is closely associated with environments where *M. spinolai* colonies are present, using the same burrows dug by *O. degus* [57]. Their larger size and nocturnal habits may compensate their lower densities, providing a stable blood meal source in the semiarid Mediterranean ecosystem [58].

The European rabbit *O. cuniculus*, a feral invasive species in the Mediterranean ecosystem of Chile [59], represented almost 5% of the diet of *M. spinolai*, and it deserves special attention. This medium-sized mammal has been previously reported as infected by *T. cruzi* and a blood-feeding source in the diet of *M. spinolai* from different populations [26–28, 60]. It is reported as a feeding source particularly for fifth-instar nymphs and adults of *M. spinolai* [29]. Moreover, it has also been proposed as a valuable blood-feeding source, due to a positive relationship between rabbit abundance and population abundance of kissing bugs [22]. This suggests that invasive species may become reservoirs of *T. cruzi*, if susceptible to this parasite’s infection.

Three reptile species were detected in the diet of *M. spinolai*. Four lizard species have been recently described as naturally infected by *T. cruzi* [14], with at least one of them being capable of transmitting the parasite to kissing bugs. Therefore, understanding the role of reptiles in the maintenance and transmission of *T. cruzi* in the wild cycle is urgent for developing adequate predictive transmission models.

It is important to mention that 11 vertebrate species not found in our samples had been detected by NGS, Sanger sequencing, and/or high-resolution melting in *M. spinolai* populations as reported in other studies [28, 29, 31]. These species include the lizard *Liolaemus pseudolemniscatus*, the native birds *Mimus thenca* and *Systellura longirostris*, the native rodents *Abrothrix longipilis* and *Oligoryzomys longicaudatus*, the fox *Lycalopex culpaeus*, the domestic cat *Felis catus*, cattle *Bos taurus*, horse *Equus caballus*, donkey *Equus asinus*, and sheep *Ovis aries* [28, 29, 31]. It is possible that these species were part of the diet of some of our *M. spinolai* populations, but due to the random sampling method used to assess the blood meal source by NGS, they were not detected. The presence of domestic animals in one of those studies could be related to their proximity to human settlements; in our study, *M. spinolai* populations were on average > 740 m from the domicile, so a lower representation of human-associated animals was expected.

In the case of the blood-feeding sources of *M. parapatrica*, four out of the six established groups were identified. Aside from humans, all species corresponded to animal groups not associated with humans (one mammal, one bird, and one reptile species). In addition to Quiroga et al. [35], only one report [36] studied the diet of *M. parapatrica* (originally referred to as *M. spinolai* by [36]), and humans were not part of this kissing bug’s diet. However, other vertebrate species such as the snake *Tachymenis peruviana*, seabirds, and marine mammals were detected.

Regarding the blood-feeding sources of *T. infestans* from a sylvatic focus, only a few species were detected, probably as the result of sampling only one population, which has been studied intermittently since its discovery in 2003 [19]. Both synanthropic and wild mammals have been associated with *T. infestans* Chilean foci [12, 61]. In our study, only three species were found in the diet of *T. infestans*: humans and two from the group of mammals not associated with humans (*O. degus* and *O. cuniculus*). Within the few studies reporting the feeding sources of wild *T. infestans* populations from Bolivia, rodents were the most detected mammals; bird and reptile species were also represented but to a lesser extent [62].

One of the most relevant epidemiological findings of our study is that the only common species as a feeding source among the three triatomine species was *H. sapiens*, found in different proportions for each species, but always present in their diet. In *M. spinolai*, the frequency/proportion of humans in the diet was low when compared against sylvatic triatomine species from other ecosystems of South America [46, 62, 63]. On the other hand, humans represented almost 50% of the blood-feeding sources for *M. parapatrica* and *T. infestans*, placing them as the sylvatic triatomine species with the highest representation of humans in their diet compared to previous studies carried out in South America [46, 62, 63]. In Chile, *T. infestans* was the main vector of *T. cruzi* inside human dwellings during the twentieth century [40], and human has recently been reported as the most frequent feeding source for domiciliated triatomines from Argentina [8]. According to our results, humans could also be the most relevant feeding source for sylvatic *T. infestans* in Chile. This high representation of humans in their diet might be the result of *T. infestans* individuals traveling to nearby human dwellings and returning to their refuges [64]. The presence of humans in the diet of *M. parapatrica* is probably the result of triatomine invasion of fishermen’s dwellings or tourist’ tents, warning about the epidemiological risk of these sylvatic triatomine bugs in coastal areas of northern Chile [35].

Sylvatic triatomine species from Chile feed on a variety of vertebrate species, and many of them are detected here for the first time in their diet. NGS may also provide updated information on host geographical distribution. The blood-feeding sources detected by NGS included humans as a relevant part of the kissing bugs’ diet. This finding, coupled with the high frequency of *T. cruzi* infection previously reported in some populations of sylvatic triatomine species in Chile [12, 22, 35] should be cause for concern. The high participation of humans in the diet of *M. parapatrica* (48.46%) and *T. infestans* (50.69%) could be an effect of sample size, given by the lower number of sampled populations compared to other reports. On the other hand, the *M. spinolai* diet is based on a large sample size and human representation was much lower (6.47%). However, because of the wide geographical distribution and high population abundance of *M. spinolai*, we support the longstanding idea that this species should be monitored, especially considering that constant home invasions are reported to the health services from rural areas [21]. Future studies should attempt individually based triatomine diet analyses, assessing seasonal variations in the diet and evaluating associated anthropic factors to relate them with the presence of humans in the diet of sylvatic triatomines. Moreover, to assess whether there is parasite transmission occurring as a result of these host–triatomine contacts, both human populations living near triatomine foci and other potential hosts—as detected by the feeding sources—should be included in *T. cruzi* infection studies, to be able to determine whether transmission is zoonotic or enzootic.

## Conclusions

Sylvatic triatomine species present in Chile feed on a variety of vertebrate species, including 16 mammals, 14 birds, and seven reptiles. Human is part of the diet of all the analyzed triatomine species, representing 12.19% of the blood-feeding source. Our results based on vertebrate host DNA detection highlight that the sylvatic triatomine–human contact is noteworthy and provide an updated and comprehensive snapshot of the blood-feeding sources of Chilean sylvatic triatomines.

## Supplementary Information

**Additional file 1: Table S1.** Complete information for triatomine populations included in the present study.

**Additional file 2:** Table S2. Results of the BLASTn of the ASVs against the NCBI nucleotide database.

**Additional file 3: Tables S3.** Complete list of vertebrate species detected as blood-feeding source for each triatomine population after ASV<100-reads filtering.

## Data Availability

The data supporting the conclusions of this article are included within the article and its additional files. Sequence data available at the NCBI Sequence Read Archive (SRA, https://www.ncbi.nlm.nih.gov/sra), project code PRJNA936617, will be released upon acceptance.

## Abbreviations

BLAST: Basic Local Alignment Search Tool
*cytb*: Cytochrome b gene
C_t_: Cycle threshold
ELISA: Enzyme-linked immunosorbent assay
IAC: Internal amplification control
NCBI: National Center for Biotechnology Information
NGS: Next-generation sequencing
ASV: Amplicon sequence variants
PCR: Polymerase chain reaction
qPCR: Quantitative (real-time) polymerase chain reaction
