# Supplementary material for: Humans as blood-feeding sources in sylvatic triatomines of Chile unveiled by next-generation sequencing
Source: Parasit Vectors. 2023 Jul 6;16:225. doi: 10.1186/s13071-023-05841-x (PMC10327138; doi:10.1186/s13071-023-05841-x)
Supplement: Supplementary file 1 — Additional file 1: Table S1. Complete information for triatomine populations included in the present study. [file 13071_2023_5841_MOESM1_ESM.docx]

**ADDITIONAL FILE 1**

**Table S1**. Complete information for triatomine populations included in the present study. The number of *T. cruzi* infected triatomines over the total captured is shown (%). When part of the information for a specific population has been totally or partially reported, the reference is given. Information collected for the present study is mentioned as “This study”. All individuals were tested for vertebrate Cytb DNA presence.

| **Species** | **Nº Population** | **Coordinate (lat; long)** | **Year** | **DNA extraction kit^1^** | ***T. cruzi* detection^2^** | **Infected/total (%)** | **Reference** |
| --- | --- | --- | --- | --- | --- | --- | --- |
| *Mepraia* sp. | 1 | -23.437; -70.608 | 2017 | QIAGEN | Cruzi1 / Cruzi2 | 14/38 (36.8) | [32] |
| *M. parapatrica* | 2 | -25.929; -70.661 | 2018 | QIAGEN | Cruzi1 / Cruzi2 | 9/29 (31.0) | [35] |
| *M. parapatrica* | 3 | -26.153; -70.690 | 2018 | QIAGEN | Cruzi1 / Cruzi2 | 12/58 (20.7) | [35] |
| *M. spinolai* | 4 | -26.804; -69.953 | 2015 | MOBIO | 121 / 122 | 10/58 (17.2) | This study |
| *M. spinolai* | 5 | -27.894; -70.917 | 2015 | MOBIO | 121 / 122 | 95/114 (83.3) | This study |
| *M. spinolai* | 6 | -28.976; -70.186 | 2015 | MOBIO | 121 / 122 | 115/268 (42.9) | [22] |
| *M. spinolai* | 7 | -30.304; -71.510 | 2019 | QIAGEN | Cruzi1 / Cruzi2 | 5/53 (9.4) | This study |
| *M. spinolai* | 8 | -30.309; -71.507 | 2019 | QIAGEN | Cruzi1 / Cruzi2 | 7/42 (16.7) | This study |
| *M. spinolai* | 9 | -30.410; -71.499 | 2019 | QIAGEN | Cruzi1 / Cruzi2 | 0/31 (0.0) | This study |
| *M. spinolai* | 10 | -30.421; -70.850 | 2015 | MOBIO | 121 / 122 | 161/165 (97.6) | [22] |
| *M. spinolai* | 11 | -30.627; -71.664 | 2019 | QIAGEN | Cruzi1 / Cruzi2 | 5/36 (13.9) | This study |
| *M. spinolai* | 12 | -30.646; -71.655 | 2019 | QIAGEN | Cruzi1 / Cruzi2 | 1/52 (1.9) | This study |
| *M. spinolai* | 13 | -30.666; -71.653 | 2019 | QIAGEN | Cruzi1 / Cruzi2 | 13/36 (36.1) | This study |
| *M. spinolai* | 14 | -30.797; -70.590 | 2015 | MOBIO | 121 / 122 | 10/365 (2.7) | [22] |
| *M. spinolai* | 15 | -30.861; -71.345 | 2015 | MOBIO | 121 / 122 | 4/190 (2.1) | [22] |
| *M. spinolai* | 16 | -31.284; -70.998 | 2015 | MOBIO | 121 / 122 | 4/315 (1.3) | [22] |
| *M. spinolai* | 17 | -31.341; -71.234 | 2016 | MOBIO | 121 / 122 | 104/122 (85.2) | [22] |
| *M. spinolai* | 18 | -31.446; -71.018 | 2014 | MOBIO | 121 / 122 | 60/142 (42.3) | [22] |
| *M. spinolai* | 19 | -31.479; -71.087 | 2020 | QIAGEN | Cruzi1 / Cruzi2 | 278/535 (52.0) | This study |
| *M. spinolai* | 20 | -31.637; -71.064 | 2014 | MOBIO | 121 / 122 | 205/224 (91.5) | [22] |
| *M. spinolai* | 21 | -31.736; -70.881 | 2016 | MOBIO | 121 / 122 | 108/114 (94.7) | [22] |
| *M. spinolai* | 22 | -31.820; -70.931 | 2016 | MOBIO | 121 / 122 | 94/108 (87.0) | [22] |
| *M. spinolai* | 23 | -32.545; -71.132 | 2016 | MOBIO | 121 / 122 | 142/157 (90.4) | [22] |
| *M. spinolai* | 24 | -32.635; -70.685 | 2015 | MOBIO | 121 / 122 | 34/106 (32.1) | [22] |
| *M. spinolai* | 25 | -32.639; -70.743 | 2015 | MOBIO | 121 / 122 | 5/159 (3.14) | This study |
| *M. spinolai* | 26 | -32.886; -70.790 | 2015 | MOBIO | 121 / 122 | 78/116 (67.2) | [22] |
| *M. spinolai* | 27 | -32.901; -70.619 | 2015 | MOBIO | 121 / 122 | 135/143 (94.4) | [22] |
| *M. spinolai* | 28 | -32.930; -70.702 | 2016 | MOBIO | 121 / 122 | 102/103 (99.0) | [22] |
| *M. spinolai* | 29 | -32.962; -71.130 | 2015 | MOBIO | 121 / 122 | 89/130 (68.5) | [22] |
| *M. spinolai* | 30 | -33.143; -70.911 | 2016 | MOBIO | 121 / 122 | 40/121 (33.1) | [22] |
| *M. spinolai* | 31 | -33.454; -70.840 | 2016 | MOBIO | 121 / 122 | 49/103 (47.6) | [22] |
| *T. infestans* | 32 | -33.655; -70.788 | 2019 | Analytik Jena | Cruzi1 / Cruzi2 | 30/54 (55.6) | This study |

^1^MOBIO, UltraClean Tissue & Cell DNA Isolation Kit; QIAGEN, DNeasy Blood & Tissue Kit; InnuPREP DNA Mini Kit, Analytik Jena; ^2^Cruzi1: 5′-AST CGG CTG ATC GTT TTC GA-3′; Cruzi2: 5′-AAT TCC TCC AAG CAG CGG ATA-3′ [51]; 121: 5′-AAA TAATGT ACG GGK GAG ATG CAT GA-3′; 122: 5′-GGT TCG ATT GGG GTT GGT GTA ATA TA-3′ [22].
